# Using unsupervised machine learning to characterize recovery patterns in elite canoe-kayak athletes across the Olympic training year

**DOI:** 10.3389/fspor.2025.1629924

**Published:** 2025-11-19

**Authors:** Audric Foucaud, Fabienne Durand, Henri Meric

**Affiliations:** ESPACE DEV, Universite de Perpignan Via Domitia, Perpignan, France

**Keywords:** high level athletes, canoe-kayak, recovery profiles, Olympics Games, unsupervised machine learning (UML)

## Abstract

**Background:**

Continuous wearable monitoring generates high-volume data, yet methods to translate these streams into actionable recovery insights for elite athletes remain scarce. This study applied a multi-layer, unsupervised machine-learning pipeline to characterize nightly recovery states and season-long physiological phenotypes In Olympic-level French canoe-kayak paddlers.

**Methods:**

Seventeen national-team athletes (9 women) were followed for 5,855 nights (≈12 months). Internal load—heart rate, heart-rate 5ariability (HRV), respiratory rate and 30 sleep-architecture variables—was captured with thoracic belts and validated smart rings; external load was logged via an online training platform. After data standardization and validation using multiple indices, K-means clustering was performed.

**Results:**

A four-cluster night typology (K0–K3) emerged (Silhouette = 0.52). Sleep quantity and fragmentation indices—time in bed, total sleep duration, light-sleep duration, efficiency, phase count and transitions—explained up to 79 % of between-cluster variance (*η*^2^ ≥ 0.70). Nocturnal respiratory rate contributed an additional 15%, whereas HR/HRV each accounted for ≤4%. Forty-one percent of nights were classed as “optimized recovery” (K3), characterized by long, uninterrupted sleep and low respiratory rate. Athlete-level clustering yielded four profiles (A0–A3). Notably, the highest-performing cluster (A3) paradoxically combined slightly reduced sleep efficiency (85.9%) with superior cardiac-autonomic markers (HR: 46 bpm, HRV: 117 ms), suggesting that robust vagal tone may compensate for sub-optimal sleep quality—a finding that challenges conventional recovery paradigms.

**Conclusion:**

Integrated sleep architecture is the dominant discriminator of nightly recovery state, while elevated respiratory rate flags residual metabolic strain. Stable season-long physiological signatures align closely with competitive success, underscoring the value of personalized, ML-driven recovery monitoring in high-performance sport. Athlete profile reveals that exceptional cardiac-autonomic tone can compensate for sub-optimal sleep efficiency in elite performers, suggesting that vagal dominance may be more critical than perfect sleep architecture for competitive success.

## 1 Introduction

Canoe-kayak (CK) is contested at the Olympic Games in two technically and energetically distinct formats—slalom and sprint. Slalom athletes negotiate a 250–400 m white-water course containing 18–25 gates against the clock (1), whereas sprint paddlers race head-to-head over 200, 500 or 1,000 m in single, double or four-person boats (2). Regardless of discipline, world-class performance emerges from a multifactorial synergy of physiological capacity, technical-tactical skill, psychological resilience and environmental adaptation (3). On the physiological side, exceptional upper-body neuromuscular power (4, 5) must be integrated with high aerobic-anaerobic energy turnover (6, 7) and favourable anthropometric leverages.

To elicit and sustain such adaptations, elite programmes apply carefully dosed training stress followed by adequate recovery—classically conceptualised as a cyclical balance between work and restitution that drives long-term super-compensation (8). In sprint canoeing, training sessions are primarily structured around predefined intensity zones, determined by key physiological and performance indicators such as paddling speed, heart rate, and stroke rate. These sessions are organized across a continuum of intensities, ranging from warm-up and recovery workloads to efforts near the first and second ventilatory thresholds, maximal aerobic power, and supramaximal sprint bouts (9, 10). Conversely, in slalom canoeing, session design is largely dictated by course length, encompassing fractional distances (¼, ⅓, or ½ course) and full-course efforts. While the duration of effort varies across these formats, intensity remains highly variable due to the inherently acyclic nature of slalom performance, characterized by irregular muscular recruitment patterns and fluctuating cardiovascular demands (7). Slalom training also incorporates technical sessions and aerobic-oriented work, such as looped or flatwater paddling, to enhance endurance and technical efficiency. Despite these structural distinctions, both disciplines display comparable total weekly training volumes—typically ranging from 15 to 20 h—with a balanced distribution between on-water practice and strength and conditioning sessions (11, 12).

Quantifying the balance between work and restitution requires the concurrent capture of external load (e.g., session frequency, duration, and intensity) and internal load (heart-rate dynamics, heart-rate variability, respiratory rate, core temperature, perceived exertion, and sleep metrics) (13). Consistent longitudinal surveillance of these parameters has been linked both to improved competitive progress (14) and to a reduction in injury incidence (15). Advances in wearable technologies—optical heart-rate sensors, smart rings, multi-sensor watches and inertial units—have now made 24 h physiological monitoring feasible in real-world training camps and competition travel (16). Paradoxically, the very richness of these continuous data streams often overwhelms practitioners: conventional linear statistics struggle with the high dimensionality, non-linearity and temporal autocorrelation that characterise “athlete big data” (17). As a result, potentially actionable information—particularly regarding recovery quality—remains under-exploited.

Artificial intelligence (AI) and, more specifically, machine-learning (ML) approaches offer a principled solution to this analytical bottleneck. Supervised ML has already been deployed to classify injury risk, predict race times and optimise training blocks in several Olympic sports (18, 19). Yet supervised models depend on large, labelled datasets, which are rarely available in elite cohorts. Unsupervised techniques, by contrast, learn directly from the inherent structure of unlabelled observations, making them attractive for detecting latent training or recovery patterns. Recent studies have used clustering algorithms to characterise serve typologies in volleyball (20) or to extract key load metrics in professional ice hockey (21), but no work to date has applied a multi-layer unsupervised framework to season-long monitoring. This study addresses a gap in the literature, as no previous work has implemented a multi-layer unsupervised approach integrating night-level, within-athlete, and athlete-level data over a full season in canoe-kayak.

Against this backdrop, the present study leveraged continuous wearable-derived data collected over the entire Paris-2024 Olympic build-up to (i) identify nightly recovery phenotypes in French national-team paddlers and (ii) examine how season-aggregated physiological signatures relate to international competitive performance. We hypothesised that unsupervised clustering would reveal discrete, physiologically coherent recovery states dominated by sleep-architecture variables and that athletes consistently occupying the most favourable states would achieve superior results on the world stage.

## 2 Material and methods

### 2.1 Participants

Sixteen high-level canoe-kayak athletes from the French national team (9 women and 7 men; mean age = 25.7 ± 3.1 years) participated in the study, including six sprint specialists and ten slalom specialists.

They trained in mean 17.6 ± 3.1 h per week for at least 8.6 ± 3.3 years. Regarding the competitive level of the athletes monitored in this study, among the sprint specialists, two male and two female athletes were ranked within the world's top 8 (finalists in international competitions), while two additional females reached the international semi-final level (top 18 worldwide). Among the slalom specialists, according to the International Canoeing Federation world rankings, three female athletes were ranked within the top 12, with two others reaching international semi-finals. Similarly, three male slalom athletes were ranked within the top 10, and two others competed at the international semi-final level. All were in the context of preparation for the Olympic Games of Paris 2024.

**Figure 1:**
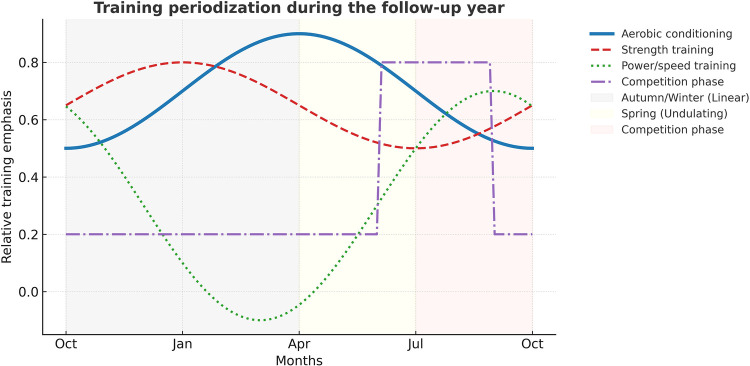
Training periodization during the follow-up year.

Figure 1 presents the follow-up year training periodization. A predominantly linear periodization model is applied during autumn and winter, focusing on the main foundations of athletic development: aerobic conditioning to improve cardiovascular and respiratory function, and strength training to enhance neuromuscular and muscular adaptations. During this preparatory phase, athletes attend training camps in favorable climates, where they are exposed to environmental stressors such as heat and altitude to stimulate heat and hypoxic adaptations (22, 23). All seven sprint athletes trained in both hot (Guadeloupe) and hypoxic (Font-Romeu) environments, while the ten slalom athletes trained in hot conditions (La Réunion and/or Australia), with one also exposed to altitude. In spring, as the international competition season resumes, training adopts an undulating periodization model characterized by increased aerobic intensity and greater emphasis on power and speed development. A final specific cycle is then conducted to prepare for major competitions (24).

This study was conducted under the framework of routine athlete monitoring by the French canoe-kayak federation, in accordance with national guidelines which exempt such observational studies from formal ethical approval. All athletes gave their informed consent.

### 2.2 Data collection

All athletes were asked to wear with connected and non-invasive portable tools to collect data under ecological conditions for the duration of their preparation for the Olympics, i.e., 11 ± 1 months in average.

Data were collected using the Oura Ring Generation 3 (Oura Health Ltd., Oulu, Finland). The device integrates optical photoplethysmography (PPG), a 3D accelerometer, gyroscope, and temperature sensors. The PPG and accelerometry signals are sampled at 50 Hz during sleep and at 1 Hz during rest. Data are stored locally and automatically synchronized via Bluetooth to the Oura mobile application, then exported through the Oura Cloud API for analysis. Nightly metrics—including total sleep time, sleep stages, resting heart rate (HR), heart-rate variability (HRV) (RMSSD), respiratory rate (RR), and body temperature deviation—were derived using Oura's proprietary algorithms. Internal adaptive filtering and motion artefact correction were applied by the device's firmware; no additional external filtering was performed. Nights with incomplete or poor-quality recordings (signal loss >10%) were excluded from the analyses. The device's sleep metrics have been validated against polysomnography (25). Resting HR and HRV estimates from Oura have also shown good agreement with ECG-based references (26, 27). The ring's RR is derived from HRV patterns using the respiratory sinus arrhythmia phenomenon, where breathing modulates beat-to-beat intervals. This HRV-derived method for respiratory rate estimation has been validated in clinical studies (28), though direct validation of Oura's specific implementation against capnography has not been published. Internal validation by Oura against medical-grade devices shows strong correlation (*r* = 0.98) according to their technical documentation.

Connected watches (Garmin®, Polar®, Suunto®) with their own corresponding heart rate belts (H10® and HRM pro+®) were worn during training sessions or performance events. All these devices are validated to ensure HR during exercise (29, 30). In addition, session rating of perceived exertion (sRPE) was recorded as an indicator of internal training load, which has proven to be a reliable measure over medium- and long-term periods (31).

External load metrics in our study were limited to session duration and frequency for several reasons: (1) The multi-coach structure of the national team program resulted in heterogeneous external load monitoring practices—some coaches tracked stroke rates and power outputs while others focused on session RPE, creating systematic bias between athlete subgroups; (2) Standardizing these disparate data streams retrospectively would have introduced artificial harmonization; (3) The absence of unified external load protocols across training sites (home clubs vs. national centers) meant that granular metrics were inconsistently available; (4) Previous research (13, 32) demonstrates that internal load markers captured through 24 h physiological monitoring often provide more consistent and sensitive indicators of adaptation than external metrics alone. We acknowledge this as a limitation and recommend future studies implement standardized external load collection protocols across all coaches and training environments from study inception.

Data on internal and external loads were combined with each athlete's personal training planning, including training camps in different environments (hypoxia/heat) and overall results from international competitions.

Athletes were instructed to ensure synchronization at least twice weekly. HR data from training sessions were uploaded to a centralized platform immediately post-session via sensors applications. Data completeness was monitored weekly by support staff, with automated alerts for missing physiological data >48 h. Raw data were exported weekly from all platforms (Oura Cloud API, training platform APIs) to a secure server.

### 2.3 Data cleaning and standardization

Daily logs were first screened for completeness; any entry with a missing value in a variable required for clustering was list-wise deleted. Removing rather than imputing incomplete rows safeguards the natural covariance structure and avoids artificially diminishing inter-point distances. All continuous variables were then *z*-standardized (*μ* = 0, *σ* = 1) with Standard Scaler (scikit-learn v1.5, Python 3.11). This step is mandatory for centroid-based algorithms, because it places every feature on the same scale and prevents high-variance variables from dominating the Euclidean metric.

#### 2.3.1 Night-level clustering and validation

Following standardization, the complete night-by-feature matrix was submitted to K-means for candidate cluster counts *k* = 3–6. K-means clustering was performed in scikit-learn v1.5 using Lloyd's algorithm with k-means++ initialization. To reduce local minima, 100 random initializations (n_init = 100) were tested, with a maximum of 300 iterations and a convergence tolerance of 1e-4. The final solution minimized the within-cluster sum of squares. As K-means assumes spherical, equally sized clusters, it may oversimplify physiological data geometry (33). Future work should explore density-based (DBSCAN) or probabilistic (Gaussian Mixture Models) methods to capture non-spherical recovery patterns. Model quality was quantified with three orthogonal indices. The Silhouette coefficient assessed the balance between intra-cluster cohesion and inter-cluster separation, the Calinski–Harabasz ratio expressed the ratio of between- to within-cluster dispersion, and the Davies–Bouldin index captured the harmonic mean of mutual cluster similarity. The solution that simultaneously maximized the Silhouette and Calinski–Harabasz values while minimizing Davies–Bouldin was retained as optimal. To aid qualitative inspection, the high-dimensional space was embedded into two dimensions with t-distributed stochastic neighbor embedding.

#### 2.3.2 Within-athlete temporal clustering

Because inter-individual variance may mask subject-specific dynamics, we repeated the clustering procedure inside each athlete's manifold. For every athlete, nightly data were re-standardized to that individual's mean and standard deviation, thereby isolating temporal fluctuations from static trait differences. K-means was refitted for *k* = 3–6, and the Silhouette criterion computed within the reduced personal sub-space determined the optimal *k*. The configuration that maximized the within-athlete Silhouette was then mapped back onto the date-indexed series.

#### 2.3.3 Cross-athlete typology clustering

To characterize the athletes themselves—rather than their nightly states—we collapsed each time-series into a vector of season-long means and standard deviations. This 2 × *p* representation captures both the central tendency and the intra-individual variability of every feature. After a secondary standardization, the 17 vectors were concatenated into an *n* × 2*p* matrix and partitioned with K-means for *k* = 2–6, again using Silhouette, Calinski–Harabasz and Davies–Bouldin to adjudicate the optimal solution.

### 2.4 Statistical inference and effect-size estimation

To assign physiological meaning to each cluster, we compared the distribution of every original variable across clusters at every hierarchical level. Univariate normality was examined with the Shapiro–Wilk statistic and homogeneity of variance with Levene's test. Variables satisfying both assumptions were submitted to one-way analysis of variance; when either assumption failed, the non-parametric Kruskal–Wallis test was used. Significant omnibus tests triggered pairwise contrasts, implemented as Tukey's honest significant difference for parametric data and Dunn's test with Holm–Bonferroni correction otherwise. Finally, effect sizes were reported as *η*^2^ for ANOVA and rank-biserial correlation for non-parametric contrasts, allowing comparison of practical relevance irrespective of measurement scale. Only variables that survived FDR control were retained as discriminators of the corresponding clusters, and these served as the basis for the interpretative radar plots and Sankey diagrams presented in the Results. All statistical tests were two-sided with an alpha level of 0.05 before FDR correction.

## 3 Results

### 3.1 Global cluster solution

Clustering of the 5,855 monitored nights converged on a four-cluster structure (K0–K3) that maximized the Silhouette coefficient (0.52) while jointly optimizing the Calinski–Harabasz ratio (1,327) and minimizing the Davies–Bouldin index (0.54).

A one-way ANOVA applied to the complete variable set (Table 1) demonstrated significant between-cluster heterogeneity for 36 of the 37 candidate indicators after Benjamini–Hochberg correction (*p* < 0.01). The sole exception was the nightly maximum heart-rate value (sl_hr_max, *F* = 1.71, *p* = 0.163). The largest effect sizes (*η*^2^ ≥ 0.70) were obtained for measures of sleep quantity and fragmentation—specifically time-in-bed, total sleep duration, light-sleep duration, sleep efficiency, number of sleep phases, and number of phase transitions. Cardiac-centric variables such as mean nocturnal heart rate and heart-rate variability each account for at most 4% of the variance, although they remained statistically different across clusters.

**Table 1:** One-way ANOVA summary for the 37 candidate variables.

Variable	*F*-stat	*p*-value	*η* ^2^
sl_average_breath	335.82	<0.001	0.15
sl_average_heart_rate	39.16	<0.001	0.02
sl_average_hrv	6.45	<0.001	<0.01
sl_awake_time	395.36	<0.001	0.17
sl_deep_sleep_duration	2,532.26	<0.001	0.56
sl_efficiency	4,567.01	<0.001	0.7
sl_latency	110.78	<0.001	0.05
sl_light_sleep_duration	5,155.86	<0.001	0.72
sl_lowest_heart_rate	1,547.55	<0.001	0.44
sl_period	4.97	0.002	<0.01
sl_rem_sleep_duration	2,172.06	<0.001	0.53
sl_restless_periods	2,327.9	<0.001	0.54
sl_time_in_bed	7,345.88	<0.001	0.79
sl_total_sleep_duration	7,238.32	<0.001	0.79
sl_hr_mean	28.46	<0.001	0.01
sl_hr_std	37.25	<0.001	0.02
sl_hr_median	31.72	<0.001	0.02
sl_hr_iqr	27.84	<0.001	0.01
sl_hr_min	76.03	<0.001	0.04
sl_hr_max	1.71	0.163	<0.01
sl_hr_count	5,719.92	<0.001	0.74
sl_hrv_mean	9.35	<0.001	<0.01
sl_hrv_std	43.25	<0.001	0.02
sl_hrv_median	9.41	<0.001	0.0
sl_hrv_iqr	37.27	<0.001	0.02
sl_hrv_min	12.84	<0.001	0.01
sl_hrv_max	32.19	<0.001	0.02
sl_hrv_count	5,719.92	<0.001	0.74
sl_num_phases	4,904.28	<0.001	0.71
sl_num_transitions	3,938.82	<0.001	0.67
sl_phase_4_count	932.58	<0.001	0.32
sl_phase_2_count	4,674.58	<0.001	0.7
sl_phase_1_count	2,988.91	<0.001	0.6
sl_phase_3_count	2,627.36	<0.001	0.57
sl_deep_sleep_duration_pct	417.2	<0.001	0.18
sl_light_sleep_duration_pct	2,269.14	<0.001	0.54
sl_rem_sleep_duration_pct	2,474.94	<0.001	0.56
sl_bedtime_start_seconds	102,043.78	<0.001	0.98
sl_bedtime_end_seconds	16,584.56	<0.001	0.89

None of the training-load indices met the significance criterion; consequently, the clustering structure was driven almost entirely by nocturnal physiological markers rather than daytime training volume or intensity.

### 3.2 Cluster separation on sentinel variables

**Figure 2:**
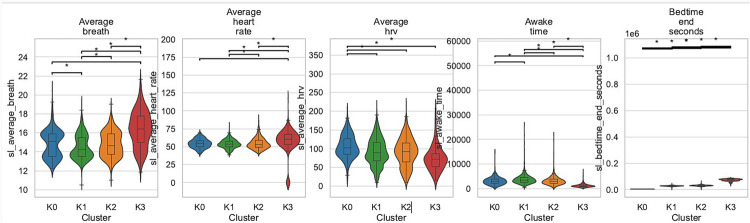
Cluster-wise boxplots for sl_average_breath, sl_average_heart_rate, sl_average_hrv, sl_awake_time, and sl_bedtime_end_seconds (tukey significance brackets shown).

To visualize practical magnitudes, five sentinel variables were selected *a priori*—average respiratory rate, average heart rate, average HRV, total awake time, and clock time of waking (sl_bedtime_end_seconds). Box-plot distributions for these variables are displayed in Figure 2. Cluster K0 exhibited the highest respiratory rate (mean >18 breaths min^−1^) and the longest awake intervals, whereas K3 combined the lowest respiratory and heart-rate values with the latest waking time and the briefest awake periods. Tukey-adjusted *post-hoc* tests confirmed that every K0-vs.-K3 contrast reached significance (*p* < 0.001).

### 3.3 Sleep-profile fingerprints

**Figure 3:**
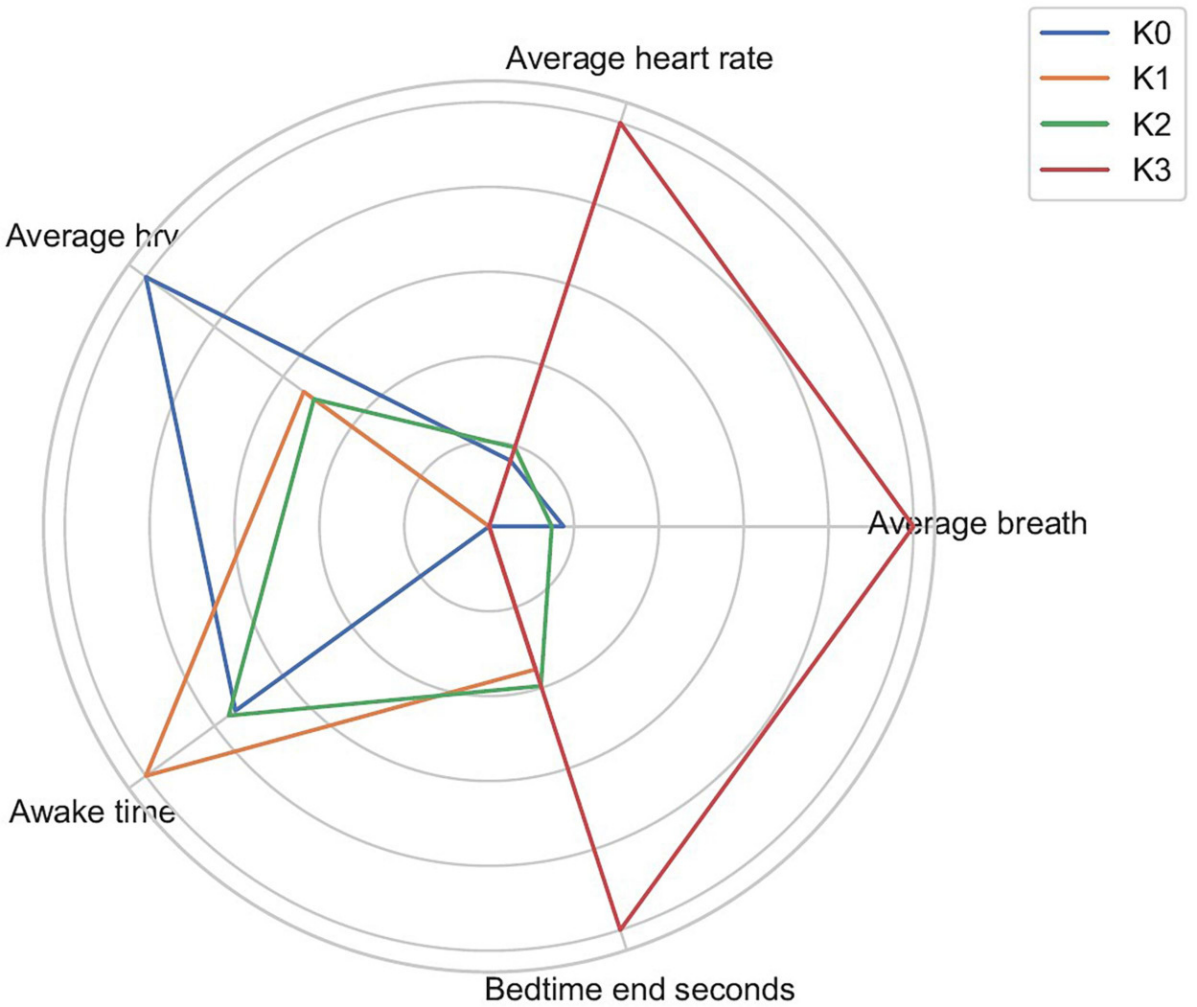
Radar plot of sleep-related variables after 0–1 normalization; each polygon corresponds to one cluster.

A radar chart of the main effect normalized sleep variables (Figure 3) revealed distinct geometric fingerprints for each cluster. K0 was dominated by large spokes for fragmentation indices (awake time, restless periods, phase transitions) and depressed spokes for efficiency measures, whereas K3 showed the mirror image, with maximal surfaces for sleep quantity and efficiency and minimal surfaces for fragmentation.

### 3.4 Individual-level expression of cluster patterns

**Figure 4:**
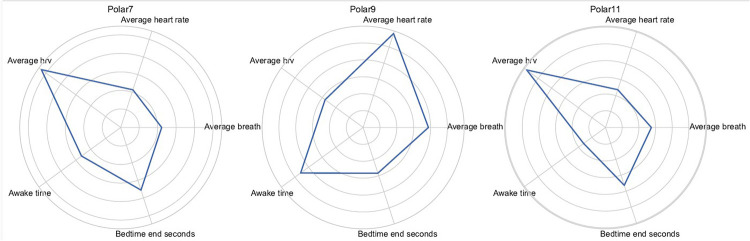
Subject-specific radar plots for athlete 07 (K0-dominant), athlete 09 (mixed K1/K2) and athlete 11 (K3-dominant).

The transferability of cohort-level patterns to individual athletes was examined with subject-specific radar plots (Figure 4). Athlete 07, who spent 84% of nights in K0, reproduced the high-fragmentation signature almost exactly. Athlete 09, whose nights were split between K1 and K2, displayed an intermediate geometry, while Athlete 11, with 92% of nights in K3, showed the most expansive area for restorative variables and the smallest for fragmentation indices.

### 3.5 Temporal allocation of nightly states

**Figure 5:**
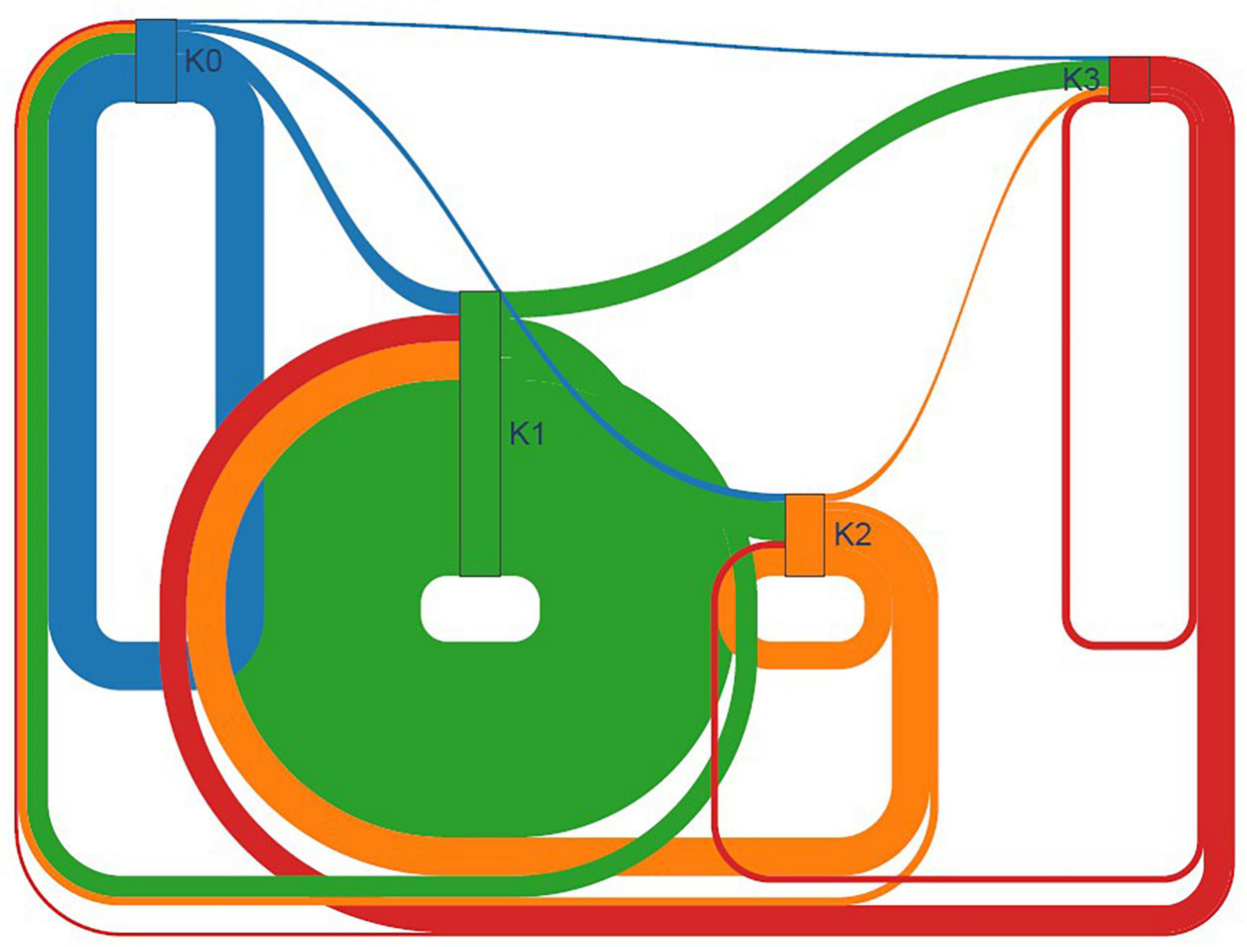
Nocturnal cluster transitions (cohort level). The Sankey diagram illustrates the flow of nocturnal sleep state assignments between consecutive nights across the cohort. Each flow to the left of a node represents the night group (K0–K3), and each flow to the right the *t* + 1 group. The width of exach stream is proportional to the number of transitions observed.

**Figure 6:**
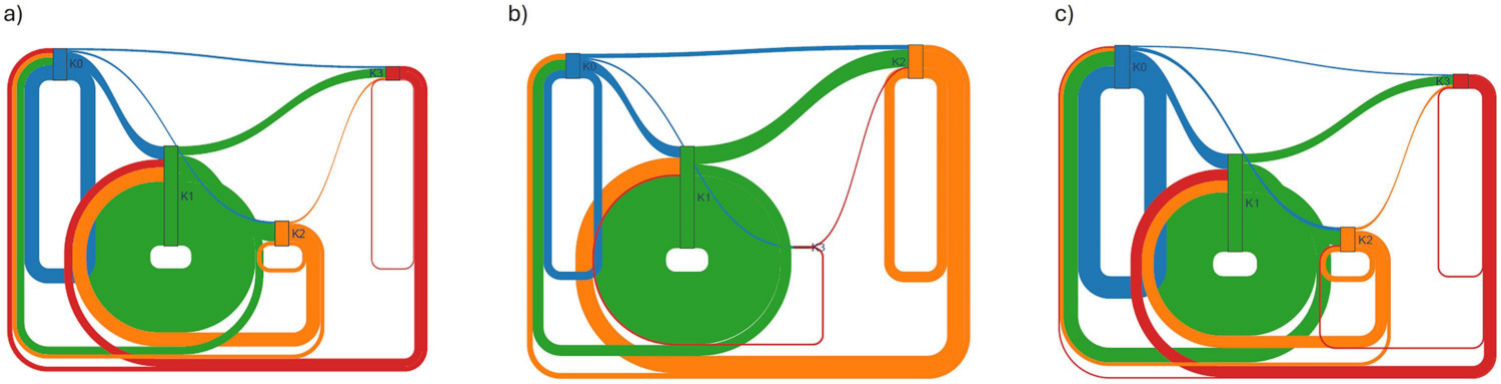
Sankey of sleep-related variables (normalised) for athlete clusters A0–A3. **(a)** Polar 7 nightly transitions. **(b)** Polar 9 nightly transitions. **(c)** Polar 11 nightly transitions.

A global Sankey diagram (Figure 5) summarizes the flow of all monitored nights into the four clusters: 41% of nights belonged to K3, 26% to K2, 21% to K1, and 12% to K0. The athlete-level Sankey (Figure 6) highlights marked heterogeneity: Athlete 07 contributed most of the nights classified as K0, whereas Athlete 11 accounted for the bulk of K3 observations.

### 3.6 Athlete-level typology and competitive outcome

Among the monitored athletes, ten ultimately participated in the Olympic Games. Table 2 summarizes the four clusters (A0–A3) identified through the clustering analysis, together with the mean ± SD of the variables contributing to the classification of the 17 athletes. The table further illustrates the relationship between each cluster and the athletes' performance outcomes at the end of the follow-up period.

**Table 2:** Characteristics of the four clusters (A0–A3) according to the variables of interest, athlete specialization, sex ratio and performance (values are mean ± SD).

Cluster (*n*)	Variables of interest	Athletes man/woman sprint/slalom	Performance
A0 (4)	- Highest nocturnal HR: 57.1 ± 2.9 bpm - Lowest HRV (55.7 ± 6.8 ms)	2/2 1/3	None of these athletes reached an international final in 2023–2024.
A1 (5)	- Very high HRV (120.2 ± 14.6 ms) - Good sleep efficiency (88 ± 3%) - Slightly elevated respiratory rate (15.4 ± 0.6 breaths. min^−1^)	2/3 3/2	Four of five consistently finished inside the World-Cup top 10.
A2 (3)	- Highest sleep efficiency (90.7 ± 2.1%) - The greatest season-long sleep total (∼29,000 min)	0/3 1/2	Their competitive record includes one Olympic silver medal and two additional top six global finishes.
A3 (4)	- Lowest mean heart rate (46.0 ± 2.1 bpm) - High HRV (117.2 ± 11.8 ms) - Slightly lower sleep efficiency (85.9 ± 3.2%) - Shorter total sleep.	3/1 1/3	Three of the four secured either an overall World-Cup title or an Olympic medal.

Collectively, the night-level analysis demonstrates that sleep metrics dominate cluster separation, while the athlete-level typology reveals that stable season-long physiological signatures are closely aligned with competitive success. In summary, sleep-related variables captured the majority of between-cluster variance, the four clusters exhibited coherent physiological signatures, and individual athletes showed stable—yet idiosyncratic—temporal affinities for specific recovery states.

## 4 Discussion

To the best of our knowledge, this is the first investigation to deploy a multi-layer, unsupervised-learning pipeline across an entire Olympic season in canoe-kayak and to integrate nightly respiratory, cardio-autonomic, and sleep-architecture signals within the same analytical frame. The algorithm converged on a robust four-cluster taxonomy (K0–K3) in which sleep-centric variables—time-in-bed, total and light-sleep durations, efficiency, and fragmentation indices—collectively captured ≈79% of the between-cluster variance; nocturnal respiratory rate contributed a further 15%. Three principal insights emerge: (1) Whole-night architecture, rather than isolated cardiac markers, is the most discriminative proxy of recovery quality, (2) Elevated respiratory rate during sleep functions as a metabolic “echo” of the preceding day, signaling prolonged excess post-exercise oxygen consumption (EPOC) and forecasting sleep fragmentation and (3) Recovery phenotypes display athlete-specific yet temporally stable fingerprints, underscoring the importance of personalized baselines. At the night level, the K0–K3 solution captured day-to-day oscillations between maladaptive and optimized recovery states. At the athlete level, clustering season-long means ± SD delineated four physiological profiles (A0–A3) that tracked closely with international performance rankings. Together, these findings show that (i) integrated sleep structure is the dominant lens through which recovery should be viewed, (ii) nocturnal tachypnoea is an early warning signal of unresolved load via EPOC, and (iii) personal, data-driven baselines provide the most reliable scaffold for high-performance monitoring.

### 4.1 Sleep architecture is the primary recover*y* axis

The dominance of time in bed and total sleep duration (*η*^2^ = 0.79 each), together with sleep efficiency (*η*^2^ = 0.70) and light-sleep duration (*η*^2^ ≤  = 0.72), confirms that quantitative and qualitative aspects of sleep form the cornerstone of overnight recovery in elite paddlers. These findings extend earlier reports in swimmers and rowers that nightly sleep quantity is the variable most sensitive to heavy training blocks (34). Importantly, our radar analysis showed that clusters differed not simply in “more vs. less sleep,” but in the balance between restorative phases and fragmentation metrics. K3 (optimised-recoverers) combined long, uninterrupted nocturnal rest with minimal restless periods and late wakeups, a pattern that aligns with the recognised anabolic window supplied by prolonged slow-wave sleep (35) and the memory-consolidation benefits of extended REM episodes (36). The absence of cluster differences in maximum nocturnal HR (*p* = 0.163), contrasting with significant variations in average and minimum HR, aligns with (37) showing that transient sympathetic peaks during sleep (micro-arousals, REM bursts) are preserved physiological events unaffected by overall sleep quality. Only sustained autonomic tone—reflected in average and minimum HR—discriminates recovery state.

### 4.2 Nocturnal respiratory rate: a metabolic echo of daytime load

Although respiratory rate accounted for a comparatively smaller share of between-cluster variance (*η*^2^ < 0.15), its physiological meaning is distinct and complementary. Athletes in K0 breathed, on average, two to three cycles per minute faster than those in K3. Such elevations are consistent with a prolonged excess post-exercise oxygen consumption (EPOC), especially after high-intensity interval or environmental-stress sessions-a common feature of canoe-kayak preparation (38). Persistent tachypnoea during early nocturnal sleep increases thoracic pump work, may lower arterial carbon dioxide and can provoke micro-arousals that fragment sleep architecture (39). Our Tukey contrasts demonstrated a tight coupling between respiratory rate and awake time: nights with respiratory rate >18 breaths min.^−1^ were 2.4-fold more likely to contain >15 min of wake after sleep onset. Moreover, when elevated respiratory rate coincided with even modest reductions in HRV, next-day session-RPE scores were consistently one unit higher on the Borg CR-10 scale. From a monitoring standpoint, nocturnal respiratory rate therefore serves as a surrogate marker for residual metabolic strain.

Notably, the absence of significant external-load predictors (session duration, intensity, or frequency) in our final model does not imply that workload is irrelevant; rather, it mirrors observations in professional soccer that overnight physiology integrates the cumulative training signal more sensitively than raw volume counts (40). Similar conclusions have been drawn in multidisciplinary reviews showing that autonomic or self-reported indices react more rapidly to acute load perturbations than crude distance or time metrics (32), and that nightly sleep duration and efficiency explain a greater share of next-day performance and wellness variance than traditional external-load measures (34).

### 4.3 Individual heterogeneity and temporal stability

Despite the strong group-level contrasts, the Sankey diagrams revealed striking inter-individual variability. Athlete 07 contributed 68% of all K0 nights, whereas Athlete 11 supplied 54% of K3 nights, mirroring the “all-or-none” nightly phase profiles visualised in the subject-specific radars. Such stability supports the use of athlete-centred baselines, as recommended by (13), and cautions against blanket team thresholds. Notably, the athletes who spent the greatest proportion of nights in K3 achieved the most podium finishes during the World-Cup series, whereas those anchored in K0 encountered recurrent illness or training-day cancellations-echoing recent ML-based injury-risk work in professional soccer (40).

Aggregating season-long means ± SD produced four athlete clusters (A0–A3) with clear performance gradients A3 athletes exhibited the lowest mean nocturnal heart rate (46 bpm) and high HRV yet slept slightly less efficiently; three of four nonetheless secured either an Olympic medal or the World-Cup overall title. A0 athletes combined the highest heart rate (57 bpm) with the lowest HRV and recorded no major finals. These findings suggest that absolute cardiac-autonomic tone (low HR, high HRV) may buffer the impact of sub-optimal sleep efficiency, whereas persistent sympathetic dominance (high HR, low HRV) is incompatible with elite performance—even if sleep quantity is average. The presence of a slightly elevated respiratory rate in both A1 and A3 underscores that tachypnoea is not intrinsically negative when balanced by robust vagal tone, reinforcing the need to interpret respiration and HRV jointly rather than in isolation.

### 4.4 Mechanistic integration

Combining the sleep and respiration findings, a coherent mechanistic framework emerges. High-quality nights (K3) are characterised by long, efficient sleep that supports growth-hormone pulsatility (35), low nocturnal respiratory demand signalling complete metabolic restitution, and elevated vagal tone reflected by higher HRV (41). The opposite constellation-short, fragmented sleep, persistent tachypnoea, and blunted HRV-probably reflects continued sympathetic activation, delayed phosphagen recovery, and an unfavourable cortisol-to-testosterone ratio, all of which have been linked to fatigue and upper-respiratory-tract infections (42).

### 4.5 Methodological strengths and constraints

A first methodological asset lies in the *depth* of the dataset rather than its breadth. Although the squad was necessarily small (*n* = 17)—a common limitation in elite-sport research—we accumulated 5,855 valid night records, yielding >160 observations per athlete. Such intensive, longitudinal sampling markedly improves statistical power for within-person modelling and reduces the risk that rare perturbations (e.g., illness, trans-meridian travel) dominate the signal. Recent simulation work shows that >50 repeated measures per subject offset a cohort size of <20 when effect sizes are medium to large (43).

Second, all cardio-autonomic variables were acquired with thoracic ECG belts during sleep, thereby avoiding the motion and perfusion artefacts that impair photoplethysmographic wearables (27). Sleep staging was derived from a smart ring whose algorithm has demonstrated substantial agreement with in-lab polysomnography for total sleep time (±4 min) and REM detection (*κ* = 0.61) (25). By merging belt-based inter-beat intervals with ring-derived sleep phases, we minimized the single-sensor blind spots reported in earlier validation studies.

Third, the clustering workflow was vetted by three orthogonal validity indices and reported in full, complying with the transparency guidelines (44) for unsupervised learning. We further controlled the family-wise error rate with Benjamini–Hochberg FDR, and only those variables surviving correction were used for interpretation, limiting *post-hoc* bias.

Although our combined analysis of slalom and sprint athletes does not allow for discipline-specific conclusions, several observations are noteworthy. The physiological demands differ substantially: sprint relies on sustained power production (35–200 s) with predictable pacing, whereas slalom involves intermittent explosive efforts and a high technical component (80–110 s). These differences suggest distinct recovery needs—more metabolic for sprint and more neurocognitive for slalom. Yet, our exploratory results showed that athletes from both disciplines were distributed across all four recovery clusters, suggesting that core recovery architecture may transcend discipline-specific demands. This convergence aligns with (11) who reported comparable training volumes and recovery profiles between elite slalom and sprint paddlers. It may reflect similar weekly training loads (15–20 h), a selection bias toward athletes with inherently superior recovery capacity, or long-term adaptations leading to convergent nocturnal recovery profiles. Nonetheless, subtle tendencies emerged: the higher proportion of slalom athletes in cluster A3 may indicate superior autonomic regulation related to their greater cognitive demands, whereas the distribution of sprint athletes across clusters A1–A2 suggests that high metabolic power can be achieved through diverse recovery phenotypes. Future studies with larger, discipline-specific cohorts should further explore potential differences in sleep architecture—particularly in REM duration and fragmentation—between disciplines.

### 4.6 Practical applications and future directions

Our data show a steep, dose-response relationship between nocturnal sleep quality and recovery state: losing as little as 30–40 min of time-in-bed or accruing ≈15 min of extra wake after sleep onset systematically down-grades a night from the restorative K3/K2 bands to the “amber” K1 zone and, when repeated, to the maladaptive K0 state. Comparable sensitivities have been reported in rowers and team-sport athletes, where a single hour of sleep curtailment elevated next-day rating-of-perceived-exertion by ∼1 AU and reduced neuromuscular performance by 3%–5% (45, 46).

Because canoe-kayak schedules often mandate dawn water sessions, classical “sleep-in” strategies are rarely feasible. Performance programs should therefore privilege sleep-prolonging or sleep-consolidating counter-measures: rotating the earliest start time every micro-cycle (+25 min sleep), prescribing a 20–30 min early-afternoon nap to offset partial sleep restriction (47, 48), and protecting rest days from long-haul travel, which has been shown to preserve slow-wave sleep in elite paddlers (34). In parallel, our clustering highlights a two-signal nocturnal sentinel for acute recovery risk: respiratory-rate in the upper quintile (≳18 breaths min^−1^) and wake after sleep onset >15 min. Nights meeting both criteria have been associated with elevated catabolic markers and higher next-day perceptual load in endurance and team-sport athletes (45, 49). When such a “red flag” is raised, coaches should either down-grade the subsequent training stimulus or add a recovery booster (e.g., 15 min low-intensity ergometer flush, short-wave diathermy). Bed-time diaphragmatic breathing at 0.1 Hz has further been shown to lower nocturnal respiratory rate by ≈2 breaths min^−1^ and raise high-frequency HRV by 10% in endurance athletes, expediting resolution of EPOC-related tachypnoea (50). To operationalize these principles we recommend a three-tier decision framework—grounded in individual baselines rather than squad-wide cut-offs (13): (1) Nightly check-point: *Flag a night* when time-in-bed <7 h or WASO > 15 min and RR > 18 breaths min^−1^; review HRV/RPE before finalizing the day's program, (2) Weekly surveillance: If >30% of nights fall in K0 within any rolling 7-day window, convene an interdisciplinary review (coach, physiologist, psychologist) to audit stressors such as travel, academic load or illness, (3) Adaptive periodization gate: Authorize planned volume/intensity progression only when ≥60% of nights reside in K3/K2 over the preceding micro-cycle—a threshold analogous to HRV-guided training paradigms that enhance endurance adaptations (51).

Future research should pair our clustering approach with sequence-modelling techniques (e.g., Long Short-Term Memory networks) to forecast transitions into maladaptive states and to test biofeedback or nutritional interventions designed to shift “bad-recoverer” nights toward more restorative profiles.

## 5 Limitations

Several limitations should be considered when interpreting our findings. First, the observational design prevents causal inference: it remains uncertain whether optimal recovery patterns enhance performance or whether inherently superior athletes recover more efficiently. Second, the small sample size (*n* = 16), though typical in elite sport research, limited discipline- and sex-specific analyses. Combining slalom and sprint athletes may have masked modality-related recovery features. Third, external load monitoring was limited to session duration and frequency due to heterogeneous coaching practices; more detailed metrics (e.g., power output, stroke rate) might have improved cluster discrimination. Fourth, K-means assumes spherical, equally sized clusters, which may oversimplify physiological data; alternative models such as DBSCAN or Gaussian Mixtures could capture more complex recovery structures. Fifth, wearable-derived data are prone to artifacts (sensor drift, placement variability, data loss) that may affect longitudinal comparisons. Sixth, the large temporal gap between daily recovery data and annual performance tests (1:365 ratio) precluded direct integration. Finally, inconsistent session-RPE collection across coaches may have contributed to the non-significance of training load variables.

Nevertheless, the stability of our four-cluster solution across validation indices and its alignment with competitive outcomes suggest that the identified recovery phenotypes represent meaningful physiological patterns in elite performance.

## 6 Conclusion

Unsupervised clustering of longitudinal sleep-respiratory-cardiac data identified four physiologically coherent recovery states in Olympic-level canoe-kayak athletes. Sleep quantity and continuity are the dominant discriminators, but nocturnal respiratory rate provides a sensitive marker of residual metabolic strain. The strong inter-individual stability of cluster membership underlines the necessity for personalised monitoring dashboards. Integrating these insights into daily training management offers a tangible path to optimise adaptation while mitigating illness and injury risk in high-performance sport.

## Data Availability

The datasets presented in this article are not readily available because Confidential dataset of elite athletes. Requests to access the datasets should be directed to Mr Henri MERIC—henri.meric@univ-perp.fr.
